# Subjective socioeconomic status: an alternative to objective socioeconomic status

**DOI:** 10.1186/s12874-023-01890-z

**Published:** 2023-03-28

**Authors:** Maryann Zhao, Chuan-Chin Huang, Milagros Mendoza, Ximena Tovar, Leonid Lecca, Megan Murray

**Affiliations:** 1 Department of Global Health and Social Medicine, Harvard Medical School, 641 Huntington Ave, Boston, MA 02115 USA; 2 Division of Global Health Equity, Brigham and Women’s Hospital, Boston, MA 02115 USA; 3 Socios En Salud, Lima, 15001 Peru

**Keywords:** Tuberculosis, Subjective socioeconomic status, Objective socioeconomic status, MacArthur Scale of Subjective Social Status, Asthma

## Abstract

**Background:**

Subjective “ladder” measurements of socio-economic status (SES) are easy-to-administer tools that ask respondents to rate their own SES, allowing them to evaluate their own material resources and determine where it places them relative to their community. Here, we sought to compare the MacArthur Scale of Subjective Social status to the WAMI, an objective measure of SES that includes data on water and sanitation, asset ownership, education, and income.

**Methods:**

Leveraging a study of 595 tuberculosis patients in Lima, Peru, we compared the MacArthur ladder score to the WAMI score using weighted Kappa scores and Spearman’s rank correlation coefficient. We identified outliers that fell outside the 95^th^ percentile and assessed the durability of the inconsistencies between scores by re-testing a subset of participants. We then used Akaike information criterion (AIC) to compare the predictability of logistic regression models evaluating the association between the two SES scoring systems and history of asthma.

**Results:**

The correlation coefficient between the MacArthur ladder and WAMI scores was 0.37 and the weighted Kappa was 0.26. The correlation coefficients differed by less than 0.04 and the Kappa ranged from 0.26 to 0.34, indicating fair agreement. When we replaced the initial MacArthur ladder scores with retest scores, the number of individuals with disagreements between the two scores decreased from 21 to 10 and the correlation coefficient and weighted Kappa both increased by at least 0.03. Lastly, we found that when we categorized WAMI and MacArthur ladder scores into three groups, both had a linear trend association with history of asthma with effect sizes and AICs that differed by less than 15% and 2 points, respectively.

**Conclusion:**

Our findings demonstrated fair agreement between the MacArthur ladder and WAMI scores. The agreement between the two SES measurements increased when they were further categorized into 3–5 categories, the form in which SES is often used in epidemiologic studies. The MacArthur score also performed similarly to WAMI in predicting a socio-economically sensitive health outcome. Researchers should consider subjective SES tools as an alternative method for measuring SES, particularly in large health studies where data collection is a burden.

**Supplementary Information:**

The online version contains supplementary material available at 10.1186/s12874-023-01890-z.

## Background

Social determinants of health result in a gradient in health outcomes that has been studied extensively in numerous contexts. Socioeconomic status (SES) can serve two distinct purposes in epidemiological studies: first as a predictor of health outcomes and secondly as a confounder that must be controlled to elucidate the relationship between health outcomes and other key determinants [1, 2]. Investigators traditionally capture SES using “objective” quantitative measures; most commonly, these include assets, income, education, and occupation. Epidemiologists have adopted composite objective SES measurements based on ownership of durables, access to services and housing characteristics, arguing that these are more reliable and easier to collect than income or consumption expenditure [3, 4]. The WAMI is one such SES index. It is composed of four parts (access to improved Water and sanitation, Asset ownership, Maternal education and household Income) and has been shown to have a stronger association with health outcomes than other composite SES indices [5]. Despite the widespread use of SES indices like the WAMI, some have argued that they are not a reliable measure of SES, resulting in different SES classifications and varying associations to health outcomes depending on which SES indicator is used [6–10].

Self-reported or “subjective” SES is an alternative measurement, which captures individuals’ perception of their own social standing relative to the community around them. Social scientists commonly use the MacArthur’s Scale of Subjective Social status (referred to as the MacArthur ladder tool henceforth), where individuals are presented with the pictorial MacArthur ladder scale and are asked to rate their socio-economic standing in relation to their community [11]. Several lines of research motivate the use of subjective SES. First, over the last two decades, researchers found that subjective indicators are associated with a range of health outcomes, including self-rated health, mental health, cardiovascular health and mortality [12–18]. Subjective SES has been shown to be independently associated with other objective indicators, to be a stronger predictor of health outcomes than objective measures, and to mediate the relationship between objective SES and health [14, 18–21]. In addition, subjective SES has been shown to be associated with health outcomes, independently of objective SES [14, 19]. Thus researchers have suggested that subjective SES reflects relevant or additional dimensions of SES that cannot be captured through objective measurements. Secondly, researchers have described self-reported SES as a comprehensive measure where individuals can judge which objective SES factors are the most important contributors to their subjective SES [12]. Third, the “averaging hypothesis” proposes that subjective SES is a more dynamic assessment since individuals can evaluate their past, current and future prospects within the context of their social and cultural environment to determine their contribution to SES [13]. In contrast, objective SES is a single snapshot in time of current resources. Moreover, researchers are able to easily administer the MacArthur ladder tool in large scale, population studies, reducing the burden of data collection.

Here, we sought to assess if a subjective SES measure is comparable to a composite objective SES and could serve as an alternative tool. Using data collected from tuberculosis (TB) patients in Lima, Peru, we estimated the correlation between the MacArthur ladder and WAMI, assessed the reliability of the MacArthur ladder over a time period of 6 to 8 months and evaluated the comparative performance of the both measurements in predicting a health outcome known to be associated with SES in this setting.

## Methods

### Participants

We embedded this investigation in an ongoing cohort study of treatment outcomes of patients, who are age 14 years or older, with TB disease. Briefly, we recruited participants when they were diagnosed with pulmonary TB disease at district health centers in a defined catchment area in Lima, Peru from October 2020 to September 2021. Enrollment took place when patients were first diagnosed, at which time we obtained demographic data and clinical samples, including socio-economic information. The participation rate of the subject population was 74%.

### Socio-economic measurements

#### Socio-demographic information

Participants completed a questionnaire that included information on race, ethnicity, education (level and years), guardian education (level and years) for minors, job status, self-reported SES using the MacArthur ladder tool, source of drinking water, sanitation facilities, housing characteristics and materials (floor, roof, wall), household size, income and asset ownership (Table 1) [11]. Income was reported as average monthly income in Peruvian soles and converted to US dollars using a conversion rate of (1 Peruvian Sol = 0.25 USD).

**Table 1:** Socio-economic status characteristics of TB patient cohort (*n* = 595)

	**All (*n* = 595)**
Female	220 (36.2%)
Age^a^	30 (23–50)
Educational Level	
No school	11 (1.9%)
Primary School	59 (9.9%)
High School	345 (58%)
Technical Studies or University	179 (30%)
Unknown	1 (0.2%)
Employed	177 (30%)
WAMI	
Improved Drinking Water	584 (98%)
Improved Sanitation	584 (98%)
Educational Years^a^	11.0 (9.5 – 13.0)
Income	
< $100–224	238 (40%)
$224–324	205 (34%)
> $324	152 (25%)
Asset Ownership	
Iron	57%
Bed	99%
Chair or Bench	96%
Sofa	61%
Cupboard	69%
Table	94%
Electric Fan	26%
Radio or Transistor	62%
Computer	45%
Television	93%
Mobile Phone	95%
Refrigerator	77%
Watch or Clock	53%
Bike	28%
Bank Account	66%

^a^median (IQR)

#### Subjective SES score

**Fig. 1:**
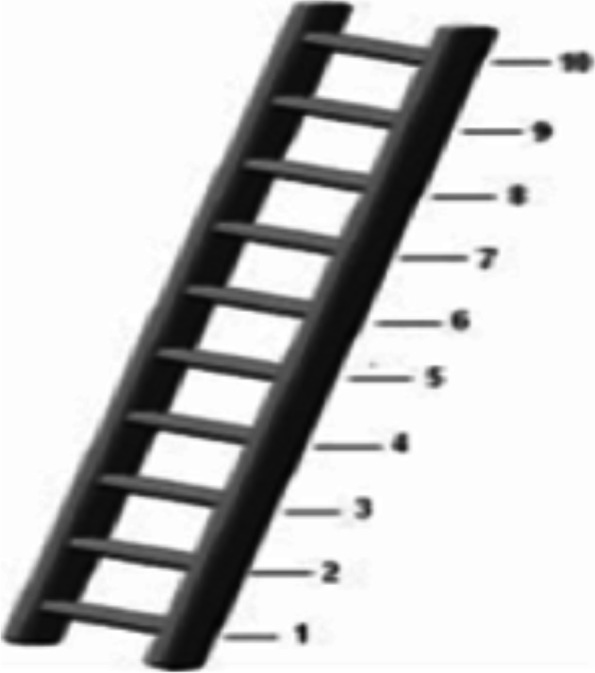
MacArthur Subjective Social Status Pictorial Ladder Tool. Pictorial ladder is shown to participants and they are asked to identify their position on the ladder where those at the top of the ladder have more money, education and better jobs and those at the bottom have less money, education, and worse jobs

For the MacArthur ladder tool, participants were shown the pictorial ladder shown in Fig. 1 and asked to identify their location using the following question:

*“Consider that the ladder that I am showing you represents the place that people occupy in society. At the top of this ladder are the people who have more money, more education and better jobs. At the bottom of the ladder are the people who have less money, less education and worse jobs (jobs with less recognition) or are unemployed.*

*The higher you consider yourself in this ladder, the closer you will be to the people who are at the top of the ladder, and the lower, closer you will be to people who find themselves at the bottom. Where would you place yourself on this ladder?"*

After 6 to 8 months, we retested 36 patients on the MacArthur ladder tool. Field workers administered the questionnaires in person or by telephone using the same initial protocol.

#### Objective SES score

We calculated the WAMI score based on responses to questions on improved Water and sanitation, durable Asset ownership, Maternal education or participant’s education and household Income. Each category is ranked from 0–8 and summed for a total out of 32 using methods described in previous studies (Table 2). The WAMI index was previously validated in a study comparing it to other approaches for measuring SES, including principal component analysis, multidimensional poverty index and education [5].

**Table 2:** WAMI scoring criteria

WAMI	Criteria	Range
Water and Sanitation	Based on WHO criteria, households with improved sources for drinking water and/or sanitation were allotted a score of 4 for each and scores were summed	0–8
Assets	Principal component analysis was performed using ownership of the 15 assets surveyed, and loading scores from the first principal component were used as the asset score. Scores were scaled to 8-points based on 9 intervals evenly spaced along the range of loading scores	0–8
Maternal Education	Educational scores were scaled based on 9 intervals evenly spaced along the range of education years of the participant if 20 years old or older, and maternal education if younger than 20 years old	0–8
Income	Participants reported their monthly household income reported using the following categories: 0 (< $100), 1 ($100–150), 2 ($150–175), 3 ($175–200), 4 ($200–225), 5 ($225–250), 6 ($250–324), 7 ($324–411), 8 (> $411)	0–8
Total	Each category was summed for the total	32

### Water

For water and sanitation, we defined improved conditions based on the World Health Organization’s guidelines [22]. Drinking water source and sanitation were considered independently and given a score of 4 each if conditions were improved.

### Assets

For durable assets, we asked participants if they owned the following 15 items: iron (either charcoal or electric), bed, chair or bench, sofa, cupboard, table, electric fan, radio or transistor, computer, television, mobile phone with paid monthly billing, refrigerator, watch or clock, bike and bank account. We performed principal component analysis on the correlation matrix of the assets, coded as binary variables [4, 23]. We used the principal component score from the first component to determine the asset score as it explains the most variance in the data (24.36%). We divided the range into 9 equal intervals to scale the scores from 0 to 8.

### Education

For participants 20 and older, we classified education level on the basis of self-reported number of years of schooling while for participants aged 14–20 who may not have completed their schooling, this was based on the number of years of education of their guardians. We divided the range of educational years into 9 equal intervals to assign a score from 0 to 8.

### Income

For income, we first sampled the precise income of 120 participants and selected the ranges for income groups to be 12.5 increment percentiles to create 9 categories. Participants chose the following category that best described their average monthly income fell into (measured in soles and converted to USD): 0 (< $100), 1 ($100–150), 2 ($150–175), 3 ($175–200), 4 ($200–225), 5 ($225–250), 6 ($250–324), 7 ($324–411), 8 (> $411).

### Data analysis

#### Agreement between WAMI and MacArthur Ladder

We first calculated the Spearman’s rank correlation coefficient between the original scales of 32-point WAMI and 10-point MacArthur ladder score. We then used a Bland–Altman plot to visually present the relationship between the two scoring systems, as well as the outliers defined as participants whose differences in their SES scores fell outside the 95th percentile on the Bland–Altman plot. Since a Bland–Altman plot requires that the paired variables have the same number of categories, we rescaled the 32-point WAMI score to an ordinal variable with 10 categories based on WAMI’s original distribution (Supplementary Table S1). Because nearly all previous studies incorporate SES in regression models as a 3–5 categorical variable, we also rescaled the 32-point WAMI and 10-point MacArthur ladder scores into 3, 4 or 5 category variables (Supplementary Fig. S2) [3, 13, 24–27]. To ensure the original distribution of scoring system was maintained during rescaling, we chose conventional cutoffs based on the histogram of the original scoring system (Supplementary Table S1). For each iteration of the two SES scoring systems, we calculated a Spearman rank correlation coefficient and a Fleiss-Cohen’s Kappa, which is a weighted kappa that penalizes greater disagreements [28, 29]. Landis and Koch’s criteria were used to interpret the Kappa statistic: a) poor: -1 to 0.20; b) fair: 0.20 to 0.40; c) moderate: 0.41 to 0.60; d) substantial: 0.61 to 0.80; and e) almost perfect: 0.81 to 1.00 [30].

#### Reassessment of the MacArthur Ladder

Although ongoing illness with TB may affect both SES measurements, the MacArthur ladder is more dependent on patients’ subjective states at the time of interview. To evaluate the durability of the inconsistencies between the MacArthur ladder and WAMI over time, we chose 14 outliers as defined above and compared these to 22 participants who were not outliers. We reassessed the MacArthur ladder score of these 36 participants 6 to 8 months after the initial survey.

#### Comparison of association between SES and Asthma

To examine whether the MacArthur ladder score is comparable to the WAMI score in an epidemiological setting, we compared the associations between the two scoring systems and the history of asthma, an outcome that previously has been shown to have an inverse linear relationship with SES [31–33]. We first evaluated the association between different categorical WAMI scores [3-, 4-, and 5- categories] and the history of asthma using a logistic regression model (adjusted for age and gender) to identify the categorical scheme that demonstrated a linear-trend association with asthma. Then, we repeated the logistic regression using the MacArthur ladder score with the same categorical scheme, followed by comparing the effect sizes and model fitting between the two logistic regressions using the Akaike information criterion (AIC). All statistical analyses were conducted in R. (https://www.r-project.org).

## Results

We enrolled 595 TB patients of whom 220 (36.2%) were female and the median age was 30 (Table 1). Nearly all participants (98%) reported improved drinking water and sanitation. 345 (58%) attended or completed high school, 179 (30%) attended or completed technical school or university, and the remaining received minimal to no education, resulting in median number of educational years of 11. For average monthly income, 238 (40%) reported themselves in the lowest bracket, 205 (34%) in the middle bracket, and 152 (25%) in the highest bracket. Some assets (bed, chair, table, television, and mobile phone) were owned by almost all of the cohort while possession of others (iron, sofa, cupboard, radio, refrigerator, watch and bank account) varied across the cohort (Table 1).

**Fig. 2:**
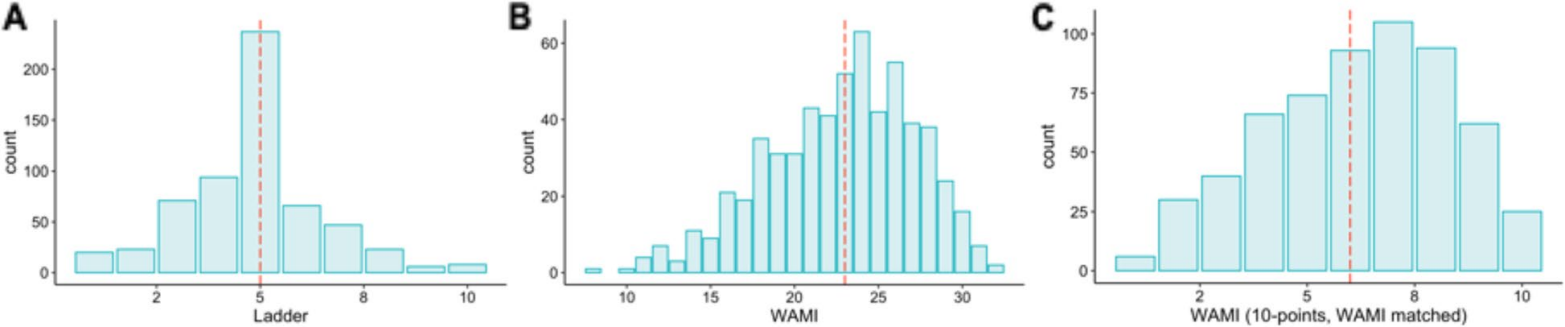
Distributions of MacArthur Ladder and WAMI Socio-economic Scores. **A** Subjective SES reported using the 10-point MacArthur ladder had a median 5 (IQR: 4–6). **B** Objective SES measured using the composite 32-point WAMI score had a median of 23 (IQR: 20–26). **C** WAMI scores rescaled to 10-points based on percentiles matching its original distribution had median of 6 (IQR: 5–8)

**Fig. 3:**
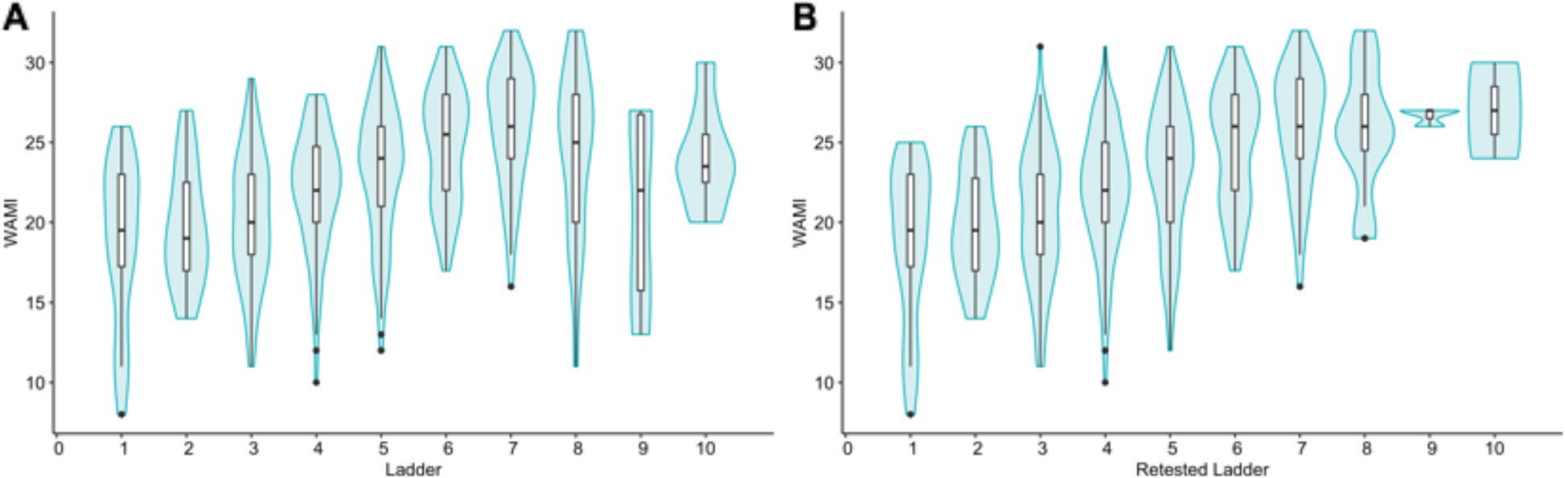
Comparison of MacArthur ladder and WAMI SES scores. **A** Violin plots with embedded boxplots display the distribution of the 32-point WAMI scores for each level of the initial MacArthur SES scale. **B** The initial MacArthur ladder scores were replaced with the retested ladder scores and plotted in a similar fashion. Alterations in the upper end of the WAMI scale resulted in a more linear increase with increasing ladder scores

**Fig. 4:**
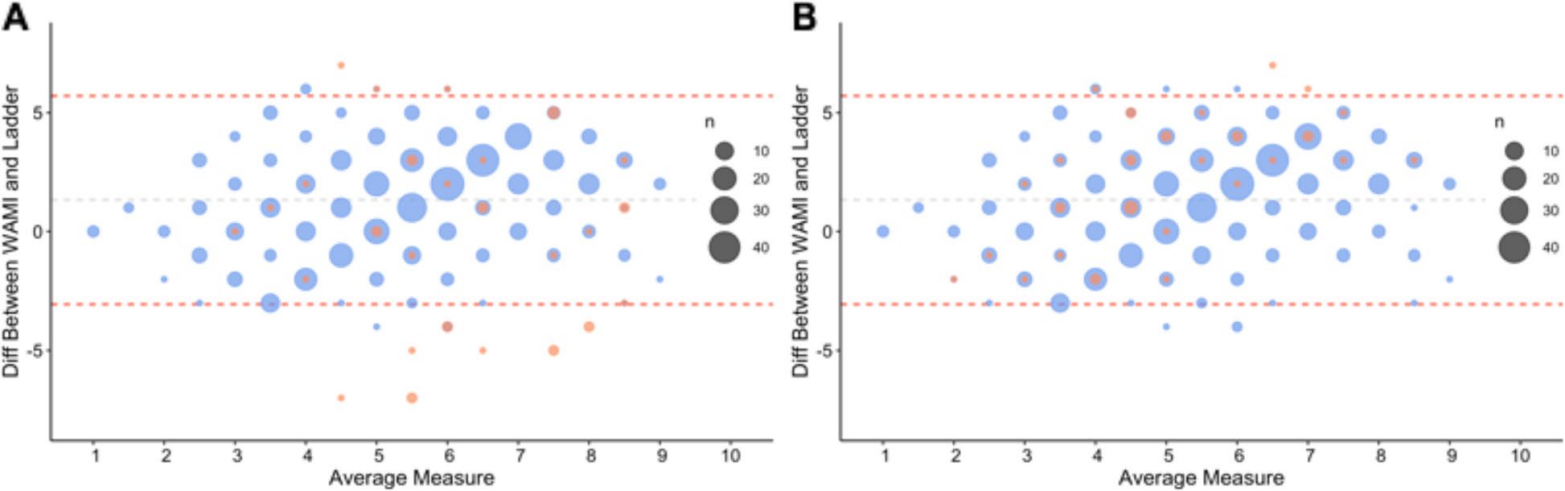
Identifying Alterations in Agreement between MacArthur Ladder and WAMI after Retest. **A** 21 individuals, whose differences between their initial MacArthur ladder scores and 10-point WAMI scores fell outside the 95^th^ percentile (red line), were identified as outliers on the Bland–Altman plot. 36 individuals (orange points) representing both outliers and non-outliers were chosen to be retested on the MacArthur ladder tool to assess for measurement error. **B** When the retested MacArthur ladder scores were used in place of the initial ladder scores for the 36 individuals, the number of outliers outside the 95^th^ percentile decreased to 10 individuals

WAMI scores ranged from 8 to 32 (out of a total of 32) with a median of 23 (IQR: 20—26) (Fig. 2). The distribution of WAMI scores was slightly left-skewed. In comparison, the median MacArthur ladder score was 5 (IQR: 4—6). Figure 3 and Table 3 show that the correlation coefficient between the original 32-point WAMI and the 10-point MacArthur ladder scores was 0.37. While some individual components of the WAMI score had lower but comparable values, including assets (*r* = 0.31), education (*r* = 0.28) and income (*r* = 0.27), the coefficient for water and sanitation was much lower (*r* = 0.038) (Table 3). When we compared the rescaled SES scores [10-, 5-, 4-, or 3-categories], the correlation coefficients between the two scores differed by less than 0.04 (Table 3). Across the different rescaling methods, the weighted Kappa statistics for the two scores ranged from 0.26 to 0.34, demonstrating fair agreement (Table 3). In the Bland–Altman plot, we found 21 (3.5%) outliers whose difference in scores fell outside the 95^th^ percentile (Fig. 4).

**Table 3:** Association between MacArthur ladder and SES indicators

WAMI	Correlation Coefficient with Ladder^a^	Correlation Coefficient with Retested Ladder^a^	Kappa with Ladder	Kappa with Retested Ladder
32-point	0.37	0.41	–	–
10-point	0.37	0.41	0.26	0.29
5-categories	0.35	0.39	0.31	0.34
4-categories	0.33	0.36	0.32	0.35
3-categories	0.34	0.38	0.34	0.37

^a^Spearman's Rank Correlation Coefficient

Once we retested participants with the MacArthur ladder scale, we replaced the original scores for all the 36 with the new scores (Fig. 3). We observed the agreement between the WAMI and MacArthur scores to improve for all categorical variables, regardless of which rescaling scheme was used. The number of outliers decreased from 21 (3.5%) to 10 (1.7%) (Fig. 4). The correlation coefficients and weighted Kappa statistics were increased by at least 0.03 and 0.03, respectively (Table 3).

After adjusting for age and gender, we found a linear trend for the association between the 3-category WAMI score and the history of asthma (but not for the 4- or 5-category WAMI score) (Table 4). Compared to those in the lowest WAMI category, individuals in the highest WAMI category had a 2.19 (95% CI 0.9 to 5.32) fold odds to report a history of asthma. In repeated logistic regression when we replaced the 3-category WAMI score by the 3-category MacArthur ladder score, we found that the linear trend association remained, the effect sizes altered by less than 15%, and the AIC changed by less than 2 (Table 4).

**Table 4:** Relationship between History of Asthma and SES^a^

SES Score (3-category)	Odds Ratios (95% CI)	AIC
**WAMI**		
Low	Reference	303.91
Middle	1.33 (0.55 to 3.24)	
High	2.19 (0.9 to 5.32)	
**MacArthur Ladder**		
Low	Reference	305.25
Middle	1.42 (0.64 to 3.15)	
High	1.87 (0.8 to 4.3)	

^a^Adjusted for age and gender, includes outliers

## Discussion

Here, we found the self-reported MacArthur ladder score for SES had fair agreement with the WAMI score, a comprehensive objective assessment of socio-economic status. When we replaced a subset of initial MacArthur scores with retested scores performed 6–8 months later, we found the agreement between WAMI and MacArthur ladder scores improved. Moreover, both 3-category scores performed similarly in predicting asthma, a health outcome known to be associated with SES [34]. Taken together, these results suggest that the MacArthur ladder score, a less cumbersome tool, can be used to replace the more detailed WAMI score with no loss in the ability to predict health outcomes or adjust for possible confounding from SES.

Our finding of a correlation of 0.34-0.41 between the ladder and WAMI scores is highly consistent with previous studies comparing objective and subjective SES measurements. These were summarized in a meta-analysis that compiled 432 associations from 357 studies which found that the ladder score was associated with a number of different “objective” scores with a mean correlation coefficient of 0.323 [35]. These results suggest that objective measures are consistently an important factor that individuals consider in self-reported SES. Given that WAMI was validated against several different objective SES measures, including a composite index calculated using PCA of household assets and the Multidimensional Poverty Index, we believe that the MacArthur ladder may serve as a suitable alternative to more complex and time-consuming SES measures. The MacArthur ladder likely captures similar components of SES as deprivation indexes, which are area-based SES measurements that include information, such as standard of living, income, education, and housing quality, that are also captured in some components of the WAMI score [36]. The MacArthur, however, is able to capture individual variability and directly measures additional subjective dimensions included in country-specific versions of the deprivation index, such as Ecuador’s index which included “poverty perception” as an indicator [37]. Interestingly, the lowest correlation between the MacArthur ladder and a WAMI component was with water and sanitation which did not vary significantly within our population; these findings suggest that area-based measurements may mask some of the nuances of individual variation in SES.

Previous qualitative analyses have reported income, material wealth, education as well as social comparison as factors respondents consider when they self-rate their SES using the ladder tool, which is consistent with our findings that assets, income and education had a correlation of 0.27–0.31 with the ladder [35]. In addition, the prevalence of asthma in Lima, Peru has previously been shown to be positively associated with SES, and the ladder and WAMI scores performed similarly in identifying this association [32].

Several mechanisms may explain why the agreement between the WAMI and the MacArthur ladder score improved when we replaced the initial MacArthur scores with those retested months later. First, while participants’ ongoing illness may impact their immediate socio-economic status when measured with either score, the MacArthur ladder score is more dependent on their subjective states. Thus, the disease states of participants at the time of enrollment may have had a greater influence on the initial MacArthur ladder scores. In addition, the field staff reported that some participants who were retested reported that they thought a lower MacArthur score indicates a higher SES during the initial screening. As a result, some of the measurement errors may have been corrected during the retests, explaining the improved agreement between WAMI and MacArthur scores when the initial scores were replaced and decreased number of outliers.

We note several limitations to our study. First, our study was conducted in a distinct population of people with lower SES in Lima, and therefore, our results may not be generalizable to a different population. However, it is reasonable to think that our findings may apply to other low- and middle-income countries where the socio-economic distributions are comparable [19, 38]. Second, our findings may be subject to selection bias if the 26% of the TB patients, who were approached by our field staff but refused to participate, had different socio-economic statuses than those who were enrolled in the study. Third, our finding that the median and mode for the MacArthur ladder was 5 raises the possibility that participants are biased to select rounded-off numbers, such as 5, which would coarsen the data. Another explanation for the increased frequency of the score 5 and the trend between the average score and the differences in SES scores observed in the Bland–Altman plots is frame-of-reference bias, which occurs when individuals are not familiar with the full range of possible socio-economic levels in their community and have minimal interaction with people in other socio-economic classes. In this case, wealthier individuals, who may be unaware of poorer individuals’ impoverished circumstances, will tend to rate themselves lower while poorer individuals tend to rate themselves higher, which was consistent with the increased differences between WAMI and the MacArthur as the mean SES score increased [3, 39].

Future studies using the MacArthur ladder may benefit from implementing methods to address scale heterogeneity and ensure interpersonal comparability of the ladder tool [40–44]. One way to address these issues is to incorporate anchoring vignettes into the questionnaires. Anchoring vignettes describe hypothetical individuals representing a specific anchor, or common, points on the ladder scale. Since the vignettes are consistent across respondents, any variation between individuals is then due to interpersonal inconsistencies, and statistical methods can be used to rescale individuals’ self-reported SES. Expanding the use of anchoring vignettes to the MacArthur ladder tool for measuring SES has yet to be explored and is a potential solution to improve the inter-person reliability and discriminatory power of subjective SES tools.

## Conclusions

Epidemiological studies have traditionally measured socio-economic status, an integral determinant of health outcomes, using objective markers and have overlooked subjective SES measurements, such as the MacArthur ladder tool, as an alternative. We demonstrated here that the MacArthur ladder had fair correlation with WAMI, an objective SES index, in categorizing patients into SES levels and performs comparably in predicting a health outcome known to be associated with SES. Given that the MacArthur ladder is simple and easy to administer, it may be considered as an alternative capable of reducing the burden of data collection in large, population-based health studies while capturing patients’ SES through a robust manner.

## Supplementary Information

**Additional file 1: Supplementary Table S1.** Rescaling Criteria for WAMI and MacArthur ladder scores.

**Additional file 2: Supplementary Fig. 1.** Scatterplot of 32-point WAMI score vs 10-point MacArthur ladder.

**Additional file 3: Supplementary Fig. 2.** Distributions of WAMI and MacArthur ladder SES scores when categorized to 3–5 groups.

## Data Availability

The datasets used and/or analysed during the current study are available from the corresponding author on reasonable request.

## Abbreviations

SES: Socioeconomic status
WAMI: Water and sanitation, Asset ownership, Maternal education and household Income score
MacArthur Ladder score: MacArthur’s Scale of Subjective Social status
TB: Tuberculosis
AIC: Akaike information criterion
