# Supplementary material for: Subjective socioeconomic status: an alternative to objective socioeconomic status
Source: BMC Med Res Methodol. 2023 Mar 28;23:73. doi: 10.1186/s12874-023-01890-z (PMC10044732; doi:10.1186/s12874-023-01890-z)
Supplement: Supplementary file 1 — Additional file 1: Supplementary Table S1. Rescaling Criteria for WAMI and MacArthur ladder scores. [file 12874_2023_1890_MOESM1_ESM.pdf]

Supplementary Table S1 | Rescaling Criteria for WAMI and MacArthur ladder scores

# of categories	WAMI Interval	MacArthur Interval
10-category		
1	8-11	--
2	12-15	--
3	16-17	--
4	18-19	--
5	20-21	--
6	22-23	--
7	24-25	--
8	26-27	--
9	28-29	--
10	30-32	--
5-category		
1	8-15	1-2
2	16-19	3-4
3	20-23	5
4	24-27	6-7
5	28-32	8-10
4-category		
1	8-17	1-3
2	18-22	4-5
3	23-27	6-7
4	28-32	8-10
3-category		
1	8-20	1-4
2	21-25	5
3	26-32	6-10
