# Supplementary material for: Subjective socioeconomic status: an alternative to objective socioeconomic status
Source: BMC Med Res Methodol. 2023 Mar 28;23:73. doi: 10.1186/s12874-023-01890-z (PMC10044732; doi:10.1186/s12874-023-01890-z)
Supplement: Supplementary file 2 — Additional file 2: Supplementary Fig. 1. Scatterplot of 32-point WAMI score vs 10-point MacArthur ladder. [file 12874_2023_1890_MOESM2_ESM.pdf]

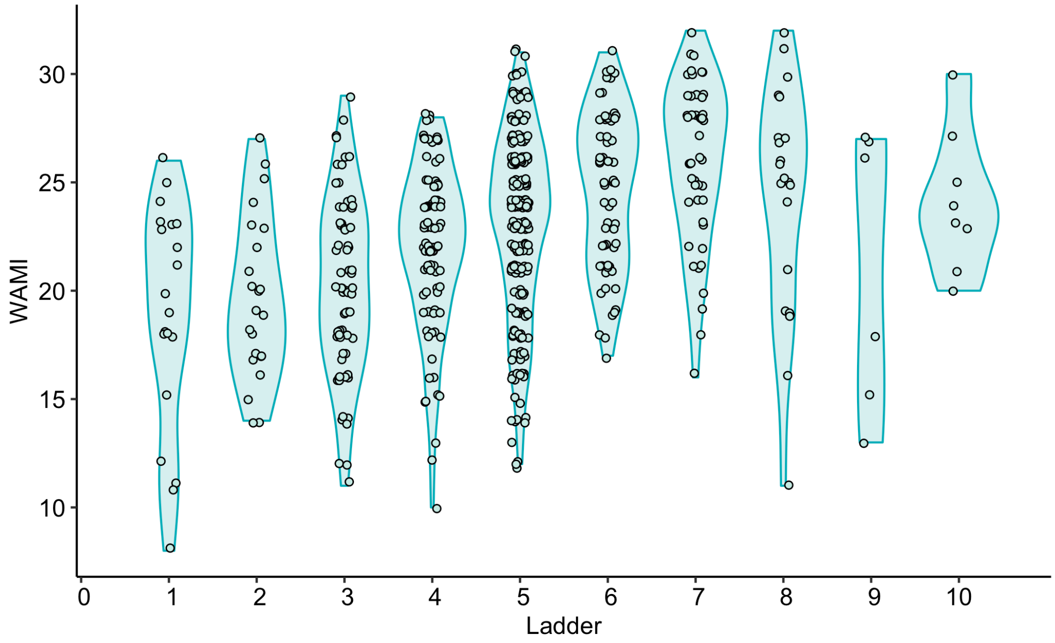

Supplementary Figure 1 | Scatterplot of 32-point WAMI score vs 10-point MacArthur ladder
The WAMI scores for each level of the MacArthur ladder can be observed on the scatterplot with the violin plots summarizing the distributions.
