# Supplementary material for: Subjective socioeconomic status: an alternative to objective socioeconomic status
Source: BMC Med Res Methodol. 2023 Mar 28;23:73. doi: 10.1186/s12874-023-01890-z (PMC10044732; doi:10.1186/s12874-023-01890-z)
Supplement: Supplementary file 3 — Additional file 3: Supplementary Fig. 2. Distributions of WAMI and MacArthur ladder SES scores when categorized to 3–5 groups. [file 12874_2023_1890_MOESM3_ESM.pdf]

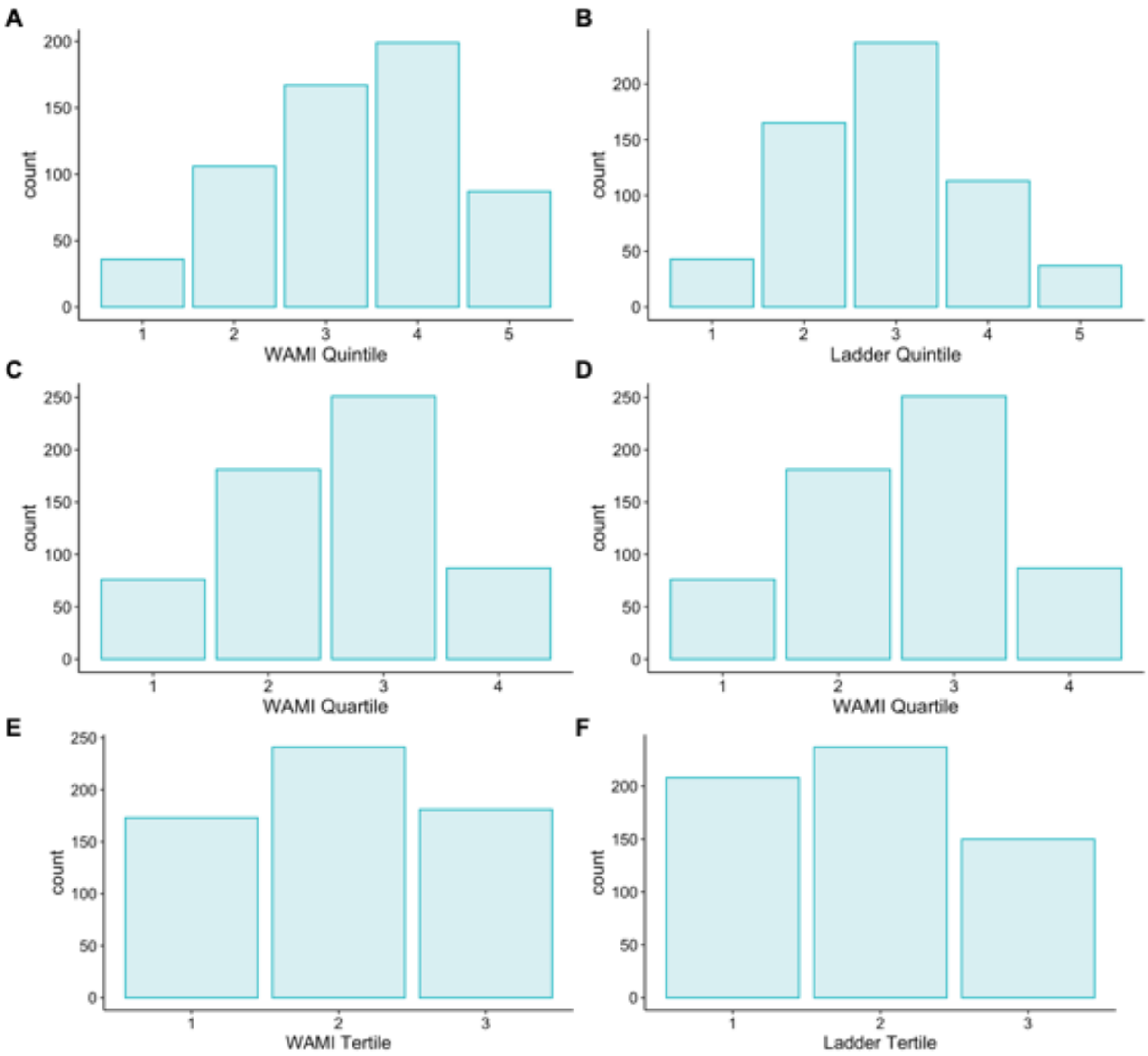

Supplementary Figure 2 | Distributions of WAMI and MacArthur ladder SES scores when categorized to 3-5 groups

A, B) WAMI and MacArthur distribution when categorized in 5 groups, C, D) WAMI and MacArthur distribution when categorized in 4 groups, E, F) WAMI and MacArthur distribution when categorized in 3 groups
